# Altered reversal and extinction learning in the DMSXL mouse model of type I myotonic dystrophy (DM1): An exploratory study

**DOI:** 10.1177/22143602251339350

**Published:** 2025-05-22

**Authors:** Sylvia Nieuwenhuis, Denys Kozakov, Kasia Kapusta, Geneviève Gourdon, Jeffrey C Glennon

**Affiliations:** 1 Department of Cognitive Neuroscience, Donders Institute for Brain Cognition and Behaviour, Radboud University Medical Center, Nijmegen, The Netherlands; 2 Department of Medical BioSciences, Radboud University Medical Center, Nijmegen, The Netherlands; 3 Sorbonne Université, Inserm, Association Institut de Myologie, Centre de recherche en Myologie, Paris, France; 4 Conway Institute of Biomolecular and Biomedical Research, School of Medicine, University College Dublin, Dublin, Ireland

**Keywords:** myotonic dystrophy type 1 (DM1), DMSXL-mice model, obsessive compulsive disorder, inflexibility behavior, autistic-like traits, appetitive extinction task, reversal learning, stimulus-action response, stimulus-reward relationship

## Abstract

**Background:**

Cognitive changes in type 1 myotonic dystrophy (DM1) have a pronounced negative effect on quality of life measures. Despite this, the neural basis of these changes is poorly understood. DM1 patients demonstrate deficits in motivation and cognitive flexibility, reflective of apathy and obsessive-compulsive / autistic-like traits.

**Objective:**

These traits can be readily assessed using reversal learning and appetitive extinction tasks. Reversal learning assesses the ability to learn following a change in a rule and can evaluate cognitive flexibility and habitual responding, while appetitive extinction assesses the ability to suppress a stimulus-action response following the change in the stimulus-reward relationship from rewarded to non-rewarded.

**Methods:**

In this study we evaluated the performance of a mouse model of DM1, the DMSXL mouse in reversal learning and appetitive extinction tasks.

**Results:**

Similar to C57/BL6 wild type (WT) mice, DMSXL mice were able to learn stimulus reward relationships, however, in the late phase of reversal learning experiment DMSXL mice demonstrated increased habit-like behavior (increased number of correct responses). Following rule switching, DMSXL mice produced an increased number of errors compared to WT and showed increased latency to deliver correct responses. In the extinction task, DMSXL mice showed the ability to more rapidly extinguish a previously rewarded response than WT mice.

**Conclusions:**

These findings constitute differences in cognitive flexibility, rule learning and motivation between DMSXL and WT mice which may inform our understanding of cognitive changes in DM1.

## Introduction

Myotonic dystrophy type 1 (DM1) is an autosomal inherited, multisystemic disorder exhibiting abnormal muscular, cardiac and brain phenotypes. It is caused by a (CTG⋅CAG)_n_-repeat length expansion within the non-coding region of *DMPK* gene.^1^ Affected genomic *DMPK* sequences upon transcription induce RNA toxicity in affected tissues. The lengths of CTG repeat expansions vary between tissues as well as from patient to patient.^2,3^ Moreover, the severity of neuromuscular symptoms coincides with a higher modal repeat number (>300). Within all three clinical presentations of DM1 (mild, classic and congenital), patients suffer from mild or severe myotonia, muscle (including cardiac) contraction abnormalities, respiratory insufficiency, cognitive impairments and early death.^1,3^

Despite the progress made in understanding of mechanisms underlying muscle dysfunction in DM1, neuromuscular complications are not the only symptoms impacting on the patient's quality of life. Cognitive symptoms (including reduced motivation, decreased IQ (Intelligence Quotient and cognitive inflexibility) allied to insomnia and fatigue have a considerably more pronounced clinical impact in DM1. Congenital and childhood-onset DM1 cases exhibit high severity of symptoms, as such, mild to severe mental retardation is often reported in affected children.^3^ DM1 neuropsychological profile is characterized by increased anxiety,^4,5^ depression,^4,6^ and anhedonia,^7,8^ while obsessive-compulsive (OCD) and autistic traits also feature prominently in this disease.^9,10^

To model cognitive changes in DM1, we studied the DMSXL mouse model. DMSXL mouse is the only animal DM1 model which carries more than 1300 CTG repeats in a human *DMPK* transgene expressed in various tissues including brain.^11^ DMSXL mice are remarkably small in size compared to their wild-type (WT) control litter mates and display muscle abnormalities (mild myotonia and general muscle weakness). Splicing abnormalities can be observed in muscle, heart and brain.^11,12^ Moreover, DMSXL showed deregulation of synaptic vesicle proteins, as well as defects in neurotransmission and behavior.^12^

In order to assess behavioral flexibility and rule-reward conditioned learning in DMSXL mice, a series of visual discrimination and reversal learning experiments were conducted, followed by a paradigm where stimulus-reward associations were extinguished (appetitive extinction). The experiments were performed in a touchscreen-based operant chamber which mimics human tablet-based cognitive testing methods, such as CANTAB,^13,14^ and enables translational comparisons relevant to rule learning impairments such as in OCD and autism. Reversal learning evaluates the ability to switch behavioral strategy in response to a shift in stimulus-reward relationships. In this case – coupling of the reinforcing reward with a previously non-rewarded stimulus. It is expected that the DMSXL mouse model displays reversal learning abilities analogous to how patients with OCD symptoms perform worse than healthy individuals in reversal learning tasks.^15–17^

In the current study, we examined the link between DM1 and compulsivity-like behavior using the DMSXL mouse model. In short, we sought to test male and female DMSXL mice and their wild type controls on 2 operant behavioral tests for cognitive flexibility and perseveration of behavior (namely reversal learning and the extinction). Breeding of these mice is associated with high mortality so we tried to assess equal number of each sex but this was not possible. As such, we examined both sexes and statistically checked to see if sex resulted in different behavioral outcomes. This study aimed to assess the differences in reversal learning and extinction performances between DMSXL and WT mice and to interpret these findings in the light of the reported behavioral inflexibility in human DM1.

## Materials and methods

### Mice

The DMSXL mice (>90% C57BL/6 background) carried 45 kb of human genomic DNA cloned from a DM1 patient as previously described (Seznec et al., 2000). DMSXL mice and control wild type (WT) mice on C57/BL6 background (n = 12 DMSXL mice, 8 male and 4 females and n = 12 wildtype mice, 8 male and 4 female) derived from breeding pairs obtained in collaboration with the group of Dr Gourdon (Centre de recherche en myologie, Paris, France). Equal numbers (*i.e.,* more females) proved difficult to breed so we phenotyped those animals available and arranged age- and sex-matched control WT mice. The mice were 8 weeks at the start of training; 16 weeks old when starting the behavioural testing and sacrified at 36 weeks. DMSXL mice were bred in-house at RadboudUMC, Nijmegen, The Netherlands and were individually housed (plexiglass cages) with crushed corncob bedding (The Andersons, Maumee, Ohio, USA), sizzle nesting material (Datesand Ltd, Manchester, UK) and an amber mouse igloo shelter (Datesand Ltd, Manchester, UK). Housing included limited food (V1244-703, SSNIFF spezialdiäten GmbH, Soest, Germany) and *ad libitum* autoclaved demineralized water diet under restriction in order to provide an appetitive incentive for operant behavior. Inside a scantainer, the animals were housed individually (Scanbur Technology, Karlslunde, Denmark) under a 12 h/ 12 h reserved light-dark cycle (lights off at 07:30 h; lights on at 19:30 h). the temperature was controlled and maintained at +/−23°C. Experimental procedures were conducted after the approval of the Animal Ethical Committee of the Radboud University (project number DEC2014-107 (breeding) and DEC2014-241 (experimentation) and followed work protocol number 150013 and project number 77175.

### Genotyping DMSXL mice

DNA was isolated from mouse ear clips using a Proteinase K treatment, followed by a isopropanol precipitation and a 70% ethanol wash. Two PCR analysis for genotyping were performed, i) one specific PCR for the human DMPK exon 3, to distinguish WT and heterozygous mice, ii) one PCR for the mouse Fbx7 gene to distinguish homozygous and heterozygous mice. Since earlier research (not yet published) showed integration from the total 45 kb insert in the human Fbx7 gene. When the insert is incorporated at both alleles in homozygous mice, using primers flanking the fragment, this will deliver no PCR product due to the large size of the insert. For the human DMPK exon 3 PCR we used next primers: Waltgour forward: 5′-GACAGTCCTAGGGTGAAGC-3′, and Waltgour reverse: 5′-TACC-TGAGGTCGAGATAGTGA-‘3. Amplification was performed using Taq2000 polymerase. The Fbx7 gene PCR was performed with the following primers: Fbx7 forward: 5′-CACCTATCCTGCCATCCC-TGTC-3′ and Fbx7 reverse: 5′-GGGACATCAGAATGG-GACATCTGC-3′.

### Food restriction and reward habituation

After the mice had completed the weaning period, they were transferred to cages for single housing and allowed 2 weeks of acclimatization to the new environment and were daily handled prior to the start of the experiments. Animals were put on a mildly restricted but not harmful diet, the animal weight loss was under strict control (85–95% of their free-feeding body weight). Prior to the start of the experiment, the food reward (strawberry milkshake, Inex N.V., Bavegem, Belgium) was introduced to the home cages in a small bowl for 2 consecutive days.

### Apparatus

Experiments were conducted in a bright room, with dim light in the boxes. The order of mice chosen for testing was randomized, however, the animals were tested in the same order during all experiments. All training was performed in Bussey-Saksida Mouse Touch Screen Chambers (Campden Instruments Ltd, Loughborough, UK) using Whisker Server (Cardinal and Aitken, 2010) and ABET II Touch software (Lafayette Instruments, Lafayette, IN, USA). These sound-attenuating, trapezoid chambers consist of fibreboard walls, a perforated stainless steel floor and a touchscreen behind a black perspex mask containing 2 – in the case of visual discrimination and reversal (VDR) – or 3 – in the case of extinction – horizontal apertures. Opposite to the touchscreen is a reward collection magazine connected to a liquid dispenser pump containing the same strawberry milkshake food reward (described above).

### Pre-training

**Figure 1.**
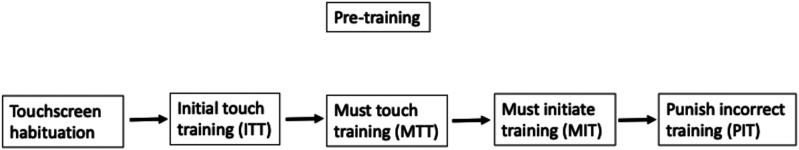
Schematic diagram of the experimental workflow to delineate the pretraining.

Mice underwent daily (5–7 times per week) training sessions, with each session lasting for a maximum of 60 min or 30 sessions (whichever was reached first). Pre-training of the animals consisted of five different stages: habituation to the touchscreen chambers, initial touch training (ITT), must touch training (MTT), must initiate training (MIT) and punish incorrect training (PIT), see Figure 1.^18^ This pre-training phase gradually shaped mice's behavior to respond to a stimulus presented in either one of the 2 response windows, which was reinforced by delivery of a liquid reward to the food magazine. The stimuli were randomly selected from an image library of 40 varied black and white shapes.

### Visual discrimination and reversal (VDR) learning paradigm

After pre-training, mice were transferred to the visual discrimination and reversal-learning task. During this task, a typical session was initiated by a nose poke into the reward collection magazine (after magazine illumination), following which a stimulus is presented within one of the response windows. Correct response to a stimulus caused reward delivery (800 ms pump activation), stimulus offset, activation of the magazine light and a brief (1 s, 3 kHz) audio stimulus (conditioned reinforcers). After reward was retrieved, a 20-s inter session interval took place. Touching the unrewarding response window during stimulus presentation was discouraged using correction sessions, which encompassed representation of the same stimulus in the same location.^19^

**Figure 2.**
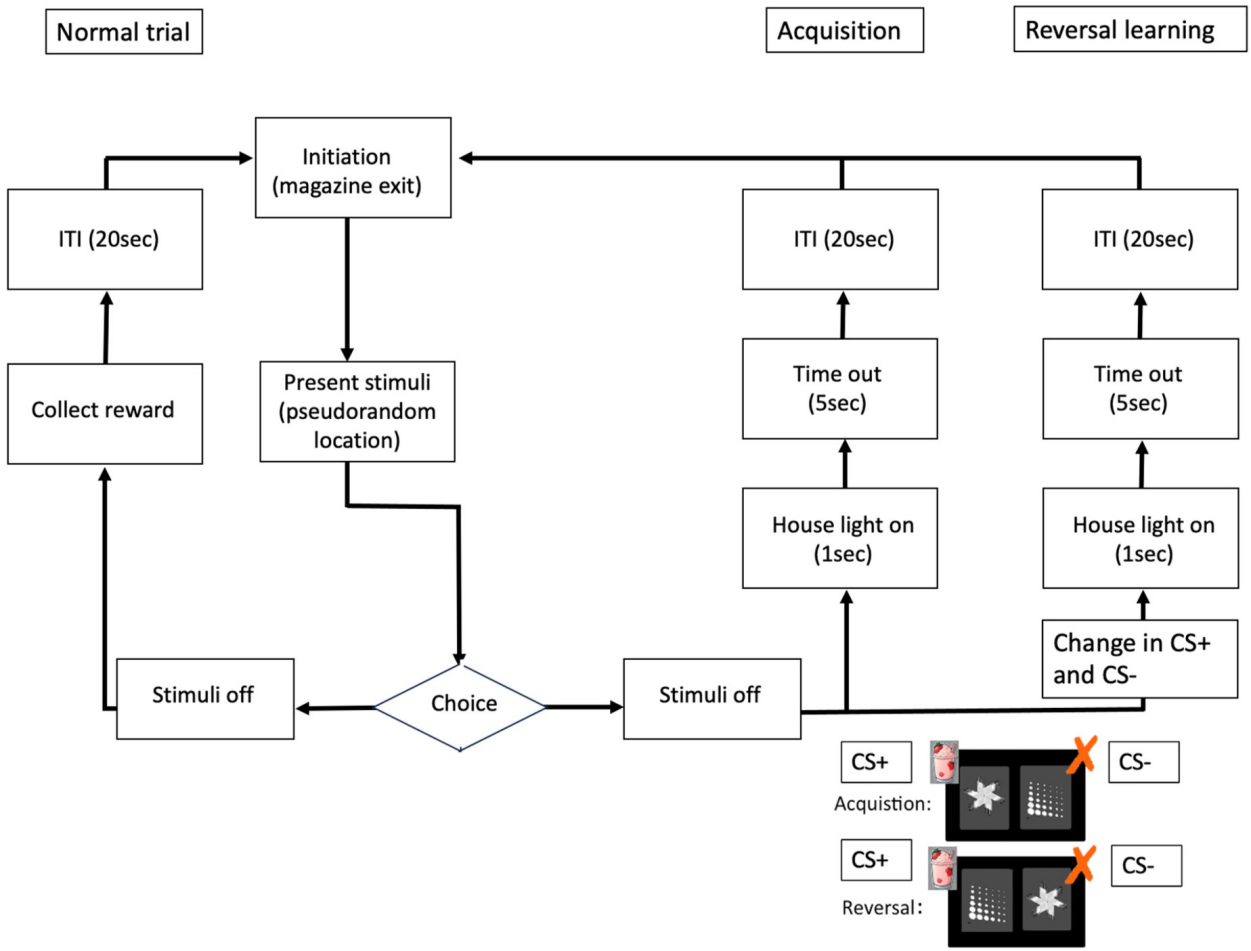
During the acquisition phase of the visual discrimination task, either a correct (conditioned rewarded) image with “fan” is on the right side or the incorrect (non-rewarded) image “marbles” is on the left side. “Fan” functioned as a conditioned stimulus (CS+) whereas the “Marbles” functioned as an unconditioned stimulus (CS-). For the Reversal learning the side on which the images were shown was randomized and the previous CS- now became the CS + and vice versa, *i.e.,* the “Marbles” became the CS + and the “Fan” became the CS-.

During the acquisition phase of the visual discrimination task, either a picture with the “Fan” or the “Marbles” functioned as a conditioned stimulus (CS+) whereas the other one was the unconditioned stimulus (CS-). Both the DMSXL and WT mice tested were able to distinguish between the 2 stimuli (Figure 2).

### Appetitive extinction

**Figure 3.**
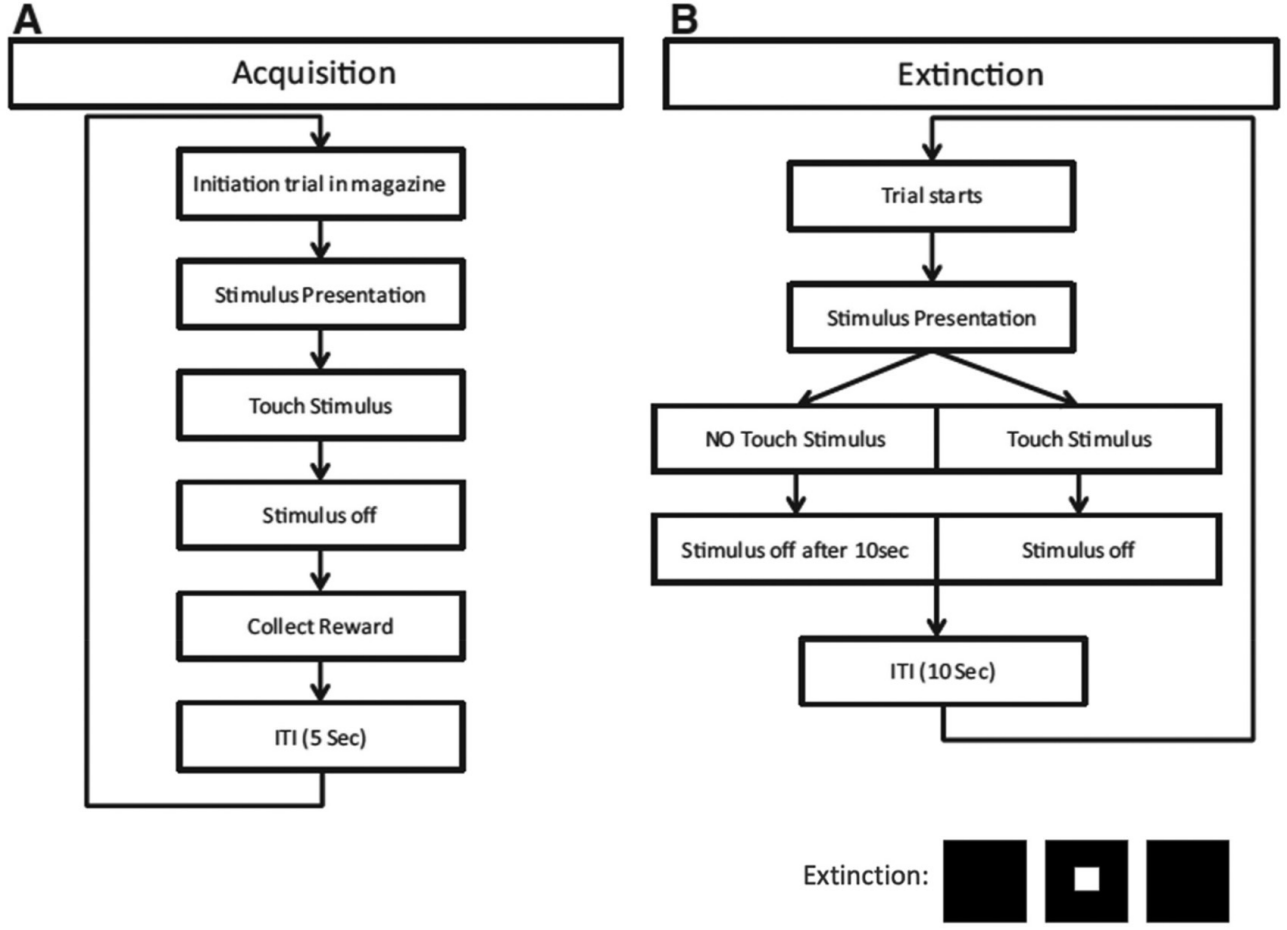
(A) in the acquisition phase, the animals are trained to collect a reward (strawberry milkshake) after correctly placing a nose poke to a stimulus (white square) and reach a stable performance criterion: 80% correct responses. (B) In the extinction phase, the stimulus (white square) is no longer rewarded.

After conclusion of the VDR paradigm, animals underwent an acquisition phase of the extinction paradigm (Figure 3). During this stage, animals were trained to respond to a rewarding white stimulus (presented in the center location of the touchscreen using a mask with three horizontal apertures). Once an animal reached the performance criterion (performing 30 sessions within 13 min for 5 consecutive days) it was moved to extinction phase of the experiment, in which the previously rewarded stimulus becomes unrewarded. A stimulus was presented for ten seconds or shorter (if the animal responded to it). The mice's performances during this phase were recorded for at least 10 consecutive days.

### Data analyses

During the acquisition phase of extinction paradigm, the total number of responses and number of correct responses were recorded, whereas during the extinction phase the number of omissions was recorded. Latencies for correct and incorrect responses, as well as reward collection were analyzed throughout the reversal and extinction sessions after outlier correction (where applicable). For outlier correction the Grubbs’ test was applied.^20^ The data was tested for normality using the Shapiro-Wilk test, after which the respective parametric or non-parametric analyses were performed. For all outcomes from mice performances in VDR behavioral tests and extinction paradigms, we analysed significance with a repeated measures ANOVA including genotype, time and the genotype*time interaction as factors and sex as covariant. We report the p-values for differences between the wild-type (WT mice) and DMSXL knock-in genotypes (DMSXL mice). The data in bar diagrams are displayed as Area Under the Curve (AUC) with mean ± SEM with *p* < 0.05 as significant, depicted as *. Where appropriate, *p* < 0.01, depicted as ** and *p* < 0.001, depicted as ***. Statistical analysis were performed in IBM SPSS Statistics 29 (IBM Corporation, Armonk, NY, USA). Graphs were created using GraphPad Prism 10 (GraphPad Software, Inc., La Jolla, CA, USA).

## Results

### Phenotype

**Figure 4.**
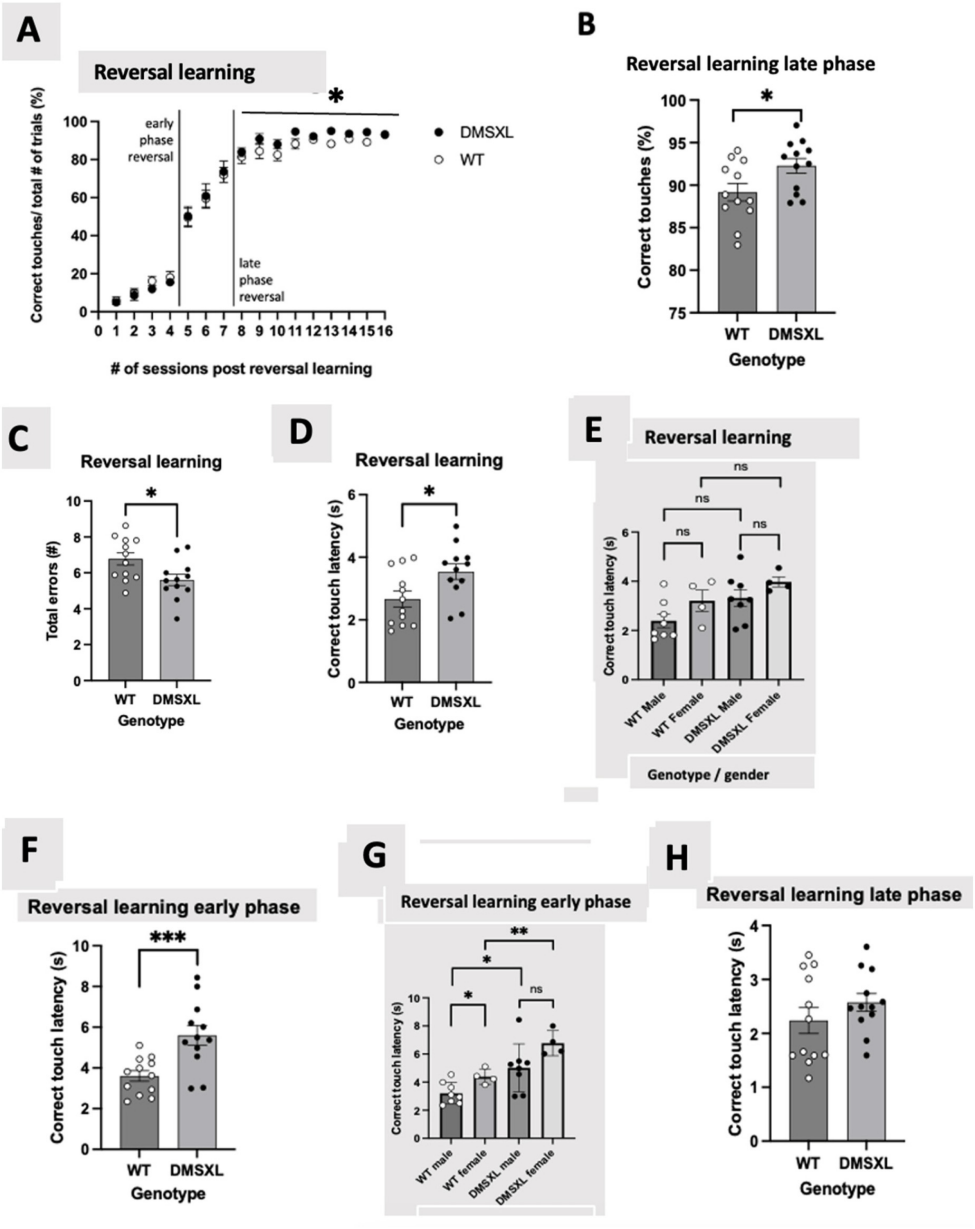
Percentage of correct touches to rewarded visual stimuli over a series of sessions in reversal phase of VDR, each bar represents an n = 12 per strain. (A) VDR reversal task is subdivided into an early phase – < 50% of correct touches, and late phase – ≥ 80% of correct responses, following reversal. No significant difference between DMSXL mice compared to WT mice (69.63 ± 7.80 vs 67.40 ± 7.32), (F = 1.007 (1,21), *p* = 0.327) was observed over the entirety phase (session 1–16) of the reversal learning task in correct touches/ total number of trials (30 trials in total) in percentage linear graph. Statistics was performed with repeated measures ANOVA. (B) During the late phase (session 8–16) of reversal learning, DMSXL mice consistently outperformed WT mice and produced a significantly higher number of correct responses compared to WT mice in correct touches / total number of trials (30 trials in total) in percentage bar-chart representation of graph A, displaying a significant difference in performance averages between DMSXL mice and WT mice (92.5 ± 1.89 vs 89.1 ± 2.02), (F = 5.138 (1,21), *p* = 0.034), depicted as *. While there is a statistical difference between WT and DMSXL mice in the late phase of reversal learning, there is no effect of sex (F = 0.081 (1,21), *p* = 0.779) as tested with a mixed model ANOVA with sex as a covariate. (C) Number of errors performed by DMSXL and WT mice throughout the entirety phase (session 1–16) of VDR reversal sessions. DMSXL mice produced a lower number of errors compared to WT mice, (data is shown as the AUC bar-chart (mean ± SEM of 5.60 ± 0.32 vs 6.78 ± 0.34), (F = 6.21 (1,21), *p* = 0.021, depicted as *). This was tested across all 16 VDR reversal sessions. Statistics was tested using a mixed model ANOVA with sex as covariate but there is no effect of sex (F = 0.379 (1,21), *p* = 0.545). Correct touch latencies of DMSXL and WT mice in the entirety (session 1–16), early (session 1–4), and late (session 8–16) of VDR reversal phase. The AUC bars represent mean ± SEM (n = 12 per strain). Statistics was tested with mixed model ANOVA with sex as covariates. (D) Throughout all VDR reversal sessions (session 1–16) DMSXL mice show increased latencies versus WT mice, to perform a correct touch (mean ± SEM of 3.54 s ± 0.53 s vs 2.66 s ± 0.33 s), (F = 7.072 (1,21), *p* = 0.015), depicted as *. There was tested for sex (F = 4.444 (1,21), *p* = 0.047), this indicates that sex was significant. (E) Checking sex significance as was shown in Figure 4A, throughout all VDR reversal sessions (session 1–16) of reversal learning, WT female mice performed a higher correct touch latency compared to WT male mice (mean ± SEM of 3.209 s ± 0.4325 s vs 2.391 s ± 0.2794 s), F = 2.697 (1,10), *p* = 0.132, depicted as ns. This indicates no significant different effect between these WT sexes. The DMSXL female mice performed a higher correct touch latency compared to DMSXL male mice (means ± SEM of 3.973 s ± 0.2014s vs 3.321 s ± 0.3402 s), F = 1.628 (1,10), *p* = 0.231, depicted as ns. This indicates no significant different effect between these DMSXL sexes. The DMSXL male mice performed a higher correct touch latency compared to WT male mice (means ± SEM of 3.321 s ± 0.3402 s vs 2.391 s ± 0.2794 s), (F = 4.460 (1,14), *p* = 0.053), depicted as ns. This indicates no significant different effect between these male genotypes. The DMSXL female mice performed a higher correct touch latency compared to WT female mice (means ± SEM of 3.973 s ± 0.2014s vs 3.209 s ± 0.4325 s), (F = 2.563 (1,6), *p* = 0.161), depicted as ns. This indicates no significant different effect between these female genotypes. Statistics was performed with repeated measures ANOVA. The AUC bars represent means ± SEM (n = 8 WT male, n = 4 WT female, n = 8 DMSXL male and n = 4 DMSXL female). (F) During early reversal phase (session 1–4), DMSXL conserve the tendency to take longer to respond (higher latencies) correctly to a stimulus than WT mice (mean ± SEM of 5.602 s ± 0.485 s vs 3.603 s ± 0.258 s), (F = 17.801 (1,21), *p* < 0.001), depicted as ***. Sex was significant (F = 8.547 (1,21), *p* = 0.008). (G) Checking sex significance as was shown in Figure 4F, in early phase (session 1–4) reversal learning, WT female mice performed a higher correct touch latency compared to WT male mice (means ± SEM of 4.392 s ± 0.271 s vs 3.208 s ± 0.274 s), (F = 7.360 (1,10), *p* = 0.022), depicted as *. This indicates a significant different effect between these WT sexes. DMSXL female mice performed a higher correct touch latency compared to DMSXL male mice (means ± SEM of 6.771 s ± 0.449 s vs 5.017 s ± 0.603 s), (F = 3.605 (1,10), *p* = 0.087), depicted as ns. This indicates no significant different effect between these DMSXL sexes. Statistics was performed with repeated measures ANOVA. The AUC bars represent means ± SEM (n = 8 WT male, n = 4 WT female, n = 8 DMSXL male and n = 4 DMSXL female). (H) Reversal learning in late phase (session 8–16), no significant difference was noted between the groups DMSXL mice versus WT mice with an AUC value (mean ± SEM of 2.575 s ± 0.165 s vs 2.238 s ± 0.240 s), (F = 1.368 (1,21), *p* = 0.255). Sex was not significant (F = 1.539 (1,21), *p* = 0.228). DMSXL male mice performed a higher correct touch latency compared to WT male mice (means ± SEM of 5.017 s ± 0.603 s vs 3.208 s ± 0.274 s), (F = 7.459 (1,14), *p* = 0.016), depicted as *. This indicates a significant different effect between these male genotypes. DMSXL female mice performed a higher correct touch latency compared to WT female mice (means ± SEM of 6.771 s ± 0.4481 s vs 4.392 s ± 0.2709 s), (F = 20.654 (1,6), *p* = 0.004), depicted as **. This indicates a significant different effect between these female genotypes. Statistics was performed with mixed model ANOVA. The AUC bars represent means ± SEM (n = 8 WT male, n = 4 WT female, n = 8 DMSXL male and n = 4 DMSXL female).

**Figure 5.**
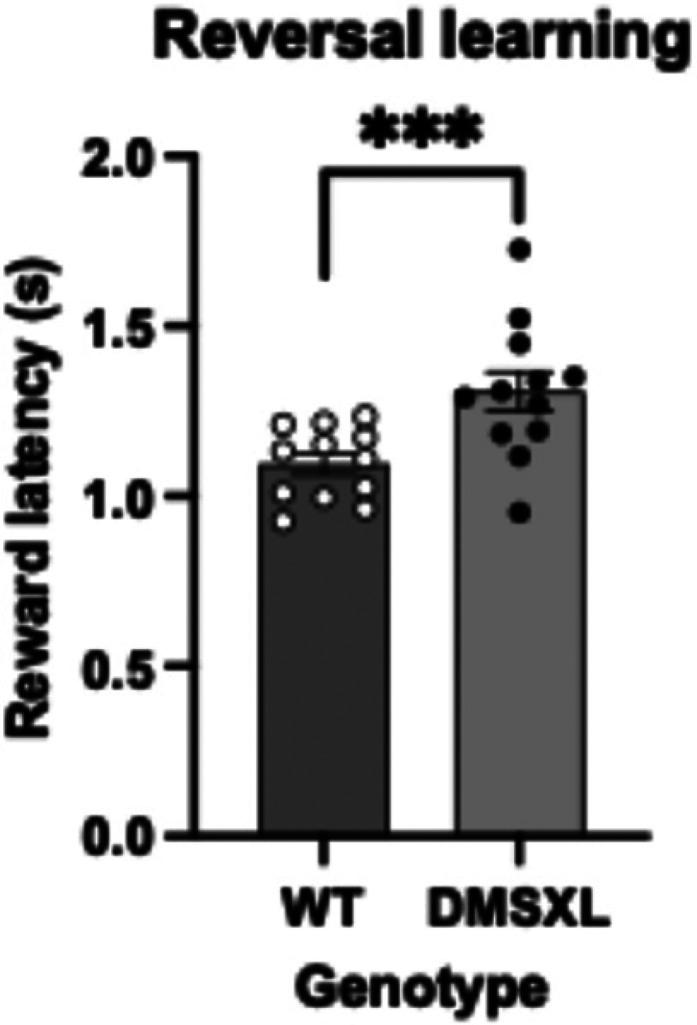
Latencies for reward collection in DMSXL and WT mice throughout VDR reversal phase. DMSXL mice display increased reward collection latencies across sessions compared to WT in a consistent manner with AUC values (mean ± SEM of 1.309 s ± 0.058 s vs 1.100 s ± 0.031 s), (F = 16.217 (1, 20), *p* < 0.001), depicted as ***. Statistical analysis was performed using a mixed model ANOVA with sex as a covariate. The bars represent means ± SEM (n = 12 per strain). There was tested between the subjects for sex and there is no effect of sex (F = 0.003 (1,20), *p* = 0.954).

DMSXL mice are known to have a developmental disturbance resulting in decreased body weight in animals at the same age / developmental time point as compared to their littermate controls, in this research body starting body weights were not measured. Due to the high mortality rate observed in homozygous mice shortly after birth, the use of males and females were not in the same proportion (males n = 8; females n = 4), since this amount of animal sexes were available from the breeding. We checked if the results could be combined across sex and since the data from the females was not significantly different from the males, we sought to report the combined groups. Unfortunately, during the behavioral performance in acquisition of extinction, for measuring the number of blank touches, one DMSXL female mouse was taken out the test (right after session 12) due to healthy problems, later this mouse died. As consequence the mouse number for the DMSXL group became n = 11, this is mentioned in the behavioral performance tests; acquisition of extinction and extinction (Figures 4 and 5 respectively).

### Pretraining

During pretraining, the DMSXL and WT mice needed a similar number of sessions to reach the criterion for each stage, indicating that they learned to correctly operate the touchscreen at a similar rate.

### Visual discrimination and reversal learning (VDR)

In the experiment both WT and DMSXL mice were able to distinguish between the 2 visual stimuli and displayed a consistent increase in proportion of the correct touches (reaching 90% accuracy) during the acquisition phase of the VDR. Both groups showed performance improvements over time with no significant difference between the two. Once all the mice have demonstrated sufficient acquisition to learn the paradigm (demonstration of >80% correct in >5 subsequent sessions), the experiment moved into the reversal phase. In the VDR trials 3 phases can be defined, namely the entirety phase consisting of session 1–16, the early phase consisting of session 1–4, and late which consist of session 8–16. Unfortunately, some recordings from the VDR, like the last 4 sessions of the extinction, for measuring the number of omissions, were lost due to technical failure, due to lower power. In the corresponding figure legend (Figure 4C) the appropriate number of trials is explicitly mentioned.

During reversal, both animal groups clearly exhibited the ability to learn and switch to a new reinforced stimulus with no consistent difference between the two groups (Figure 4A). Although both DMSXL and WT mice were able to switch strategy and learn the reversal paradigm (as shown by no difference in % of correct responses in the early phase of reversal (< 50% correct responses)), in the late reversal phase (at >80% correct responses) – DMSXL mice displayed a higher % of correct responses compared to WT (Figure 4B).

No statistical difference was noted either between genotypes or across sex in the number of correction sessions in both the acquisition and reversal phase. As shown in Figure 4E, the DMSXL mice made less mistakes, as shown by a decreased number of total errors in reversal phase in DMSXL mice compared to WT mice.

The latencies of mouse responses to stimuli related to correct touches, blank touches as well as reward collection were determined throughout both acquisition and reversal phases. Following reversal, DMSXL mice required more time to correctly respond to a stimulus in comparison to WT (Figure 4D), in the early phase of the reversal learning (Figure 1F) but not seen in the late phase (Figure 4H), suggesting that the DMSXL mice are initially slower in learning, following the switch of the rewarded stimulus. Since there is a significant effect associated with sex during early and overall reversal learning phase, we checked if there was any statistical difference between sexes underlying the difference between WT and DMSXL mice in other metrics by including sex as covariate in the analysis. No significant difference was found between WT male versus female and DMSXL male versus female in the other metrics reported.

From Figure 4A, it appears that the late phase (session 8–16) is significant different, therefore, a post hoc analysis for comparison between DMSXL and WT within the sessions is performed with an independent t-test, see Table 1 in the supplementary materials.

In the reversal learning test, after multiple testing, there were no significant differences between WT and DMSXL mice in correct responses in each session. However there is a visible trend showing that the DMSXL mice perform better compared to the WT mice.

Throughout the reversal, DMSXL mice displayed considerably higher reward collection latencies than WT (Figure 5), possibly implying a generally decreased motivation to obtaining the reward.

### Appetitive extinction

**Figure 6.**
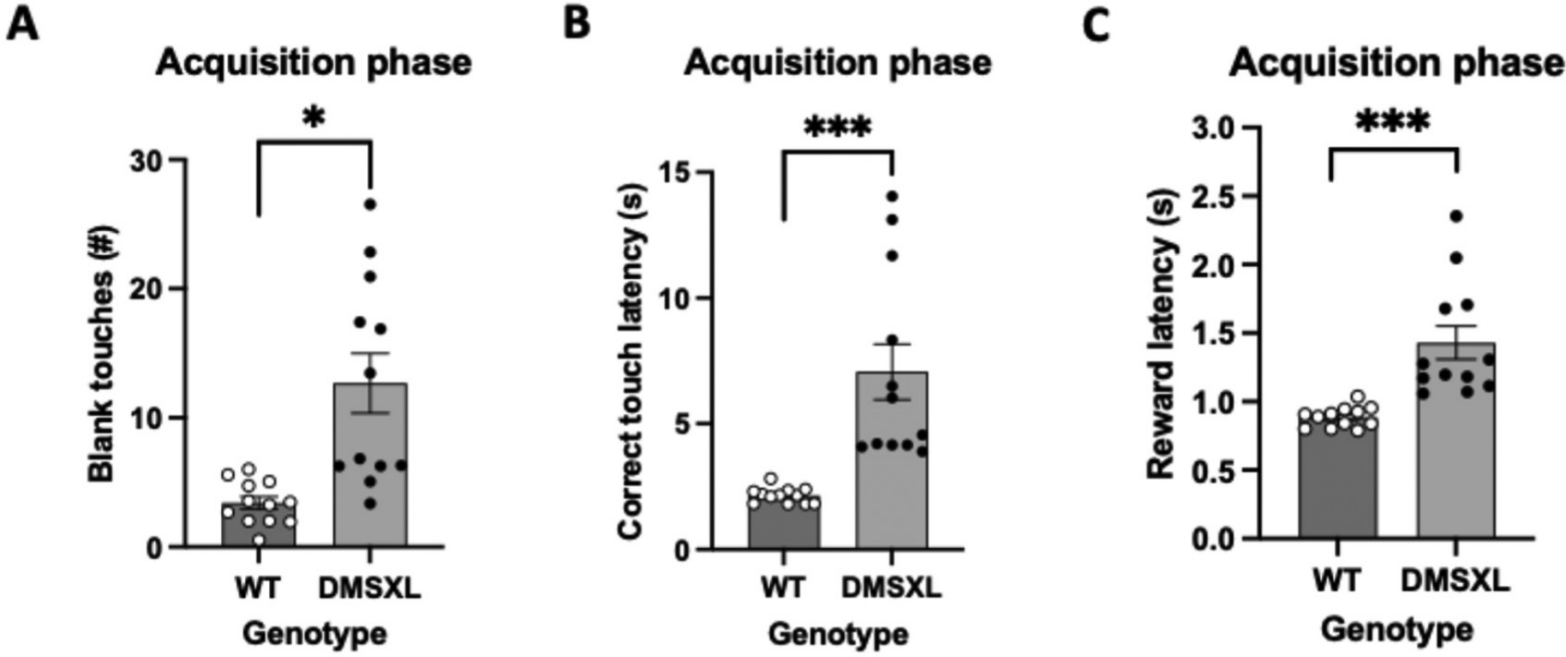
Measures of behavioral performance in acquisition of extinction in DMSXL and WT mice. Statistics, for Figures A, B and C, was performed with mixed model ANOVA with sex as covariates. The bars for WT mice represent means ± SEM with n = 12 per group and for DMSXL mice n = 11 per group. (A) Number of blank touches made by DMSXL mice in extinction acquisition phase is consistently significantly higher, results with AUC values (mean ± SEM of 12.680 ± 2.310 vs 3.404 ± 0.485), (F = 9.872 (1,20), *p* = 0.005), depicted as *. There was tested between the subjects for sex and there is no effect of sex (F = 0.363 (1,20), *p* = 0.554). (B) DMSXL mice require considerably more time to deliver a correct touch, results with AUC values (mean ± SEM of 7.063 s ± 1.101 s vs 2.143 s ± 0.088 s), (F = 18.414 (1,20), *p* < 0.001), depicted as ***. There was tested between the subjects for sex and there is no effect (F = 0.000 (1,20), *p* = 0.989). (C) In the acquisition phase of extinction task DMSXL mice are being significantly slower to pick up the reward compared to WT, results with AUC values (mean ± SEM of 1.430 s ± 0.122 s vs 0.886 s ± 0.021 s), (F = 15.660 (1,20), *p* < 0.001), depicted as ***. There was tested between the subjects for sex and there is no effect of sex (F = 0.116 (1,20), *p* = 0.737).

During acquisition phase within the appetitive extinction paradigm both mouse groups reached the criterion in an equivalent number of sessions. However, DMSXL group was observed to perform more blank touches than the control group (Figure 6A). Moreover, DMSXL mice compared to WT mice, displayed considerably higher correct touch latencies (Figure 6B) as well as reward collection latencies (Figure 6C).

**Figure 7.**
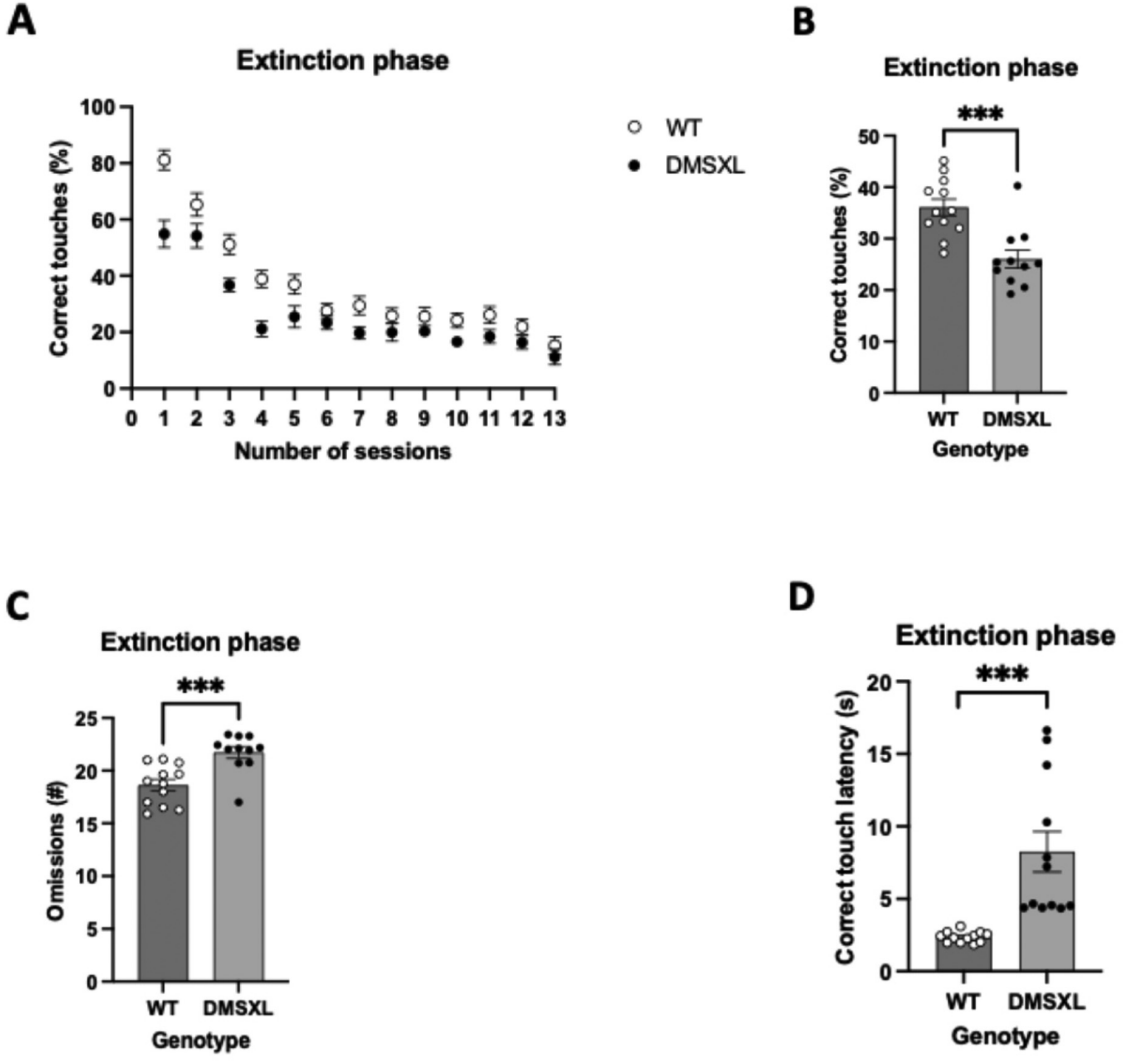
Percentage of correct responses to non-rewarded visual stimuli, number of omissions and correct touch latencies over a series of sessions within the extinction task. For all 4 panels, WT mice represent means ± SEM with n = 12 per group and for DMSXL mice n = 11 per group. For panel 7A, statistics was performed with repeated measures ANOVA. For panels 7B-7D, statistics was performed with mixed model ANOVA with sex as covariates. The bars for WT mice represent means ± SEM with n = 12 per group and for DMSXL mice n = 11 per group. (A) Temporal view of the percentage of correct touches performed by DMSXL and WT mice during the extinction task. DMSXL consistently performed less correct touches than WT in each of the consecutive session. (B) Area under the curve (AUC) bar-chart representation of graph A, displaying a significant difference in performance averages between DMSXL mice and WT mice, results with AUC values (mean ± SEM of 26.04 ± 1.750 vs 36.09 ± 1.614), (F = 17.669 (1,20), *p* < 0.001), depicted as ***. There was tested between the subjects for sex and there is no effect of sex (F = 1.680 (1,20), *p* = 0.210). (C) During 12 sessions of extinction, the number of omissions made by DMSXL mice is significantly higher compared to WT mice, results with AUC values (mean ± SEM of 18.63 ± 1.883 vs 21.73 ± 0.546), (F = 19.295 (1,20), *p* < 0.001), depicted as ***. There was tested between the subjects for sex and there is no effect (F = 1.735 (1,20), *p* = 0.203). (D) Throughout the extinction task of 16 session correct touch latencies in DMSXL mice are significantly higher than in WT mice, results with AUC values (mean ± SEM of 8.255 s ± 1.394 s vs 2.364 s ± 0.076 s), (F = 18.414 (1,20), *p* < 0.001), depicted as ***. There was tested between the subjects for sex and there is no effect (F = 0.000 (1,20), *p* = 0.989).

Following acquisition phase, in extinction DMSXL mice produced significantly less correct touches than WT (Figure 7B). An opposite trend was observed in omissions (Figure 4C) and correct touch latencies (Figure 7D), where DMSXL showed an overall lack of interest in performing the task.

For the breakdown of the different types of responses and percentages, including the total number of sessions, is presented in Table 2 in the supplementary materials.

## Discussion

Behavioral flexibility of DMSXL mice was analyzed in order to evaluate the presence of compulsive-related behaviours which are reported to be altered in human DM1.^12^ The DMSXL mouse model does have the limitation that the site of transgene insertion (reported by unpublished data to be at the Fbx7 locus) which may impact the findings. This possibility is not controlled for in the design used here given the difficulty in breeding sufficient numbers of DMSXL mice and as such this is a pilot behavioural study. Despite these caveats, the current study sought to extend our knowledge of the DMSXL phenotype using two different behavioural tasks which evaluate different aspects of compulsive behaviour namely the ability to learn and switch strategy (reversal learning) and the ability to inhibit action following a change in contingency from rewarded to non-rewarded (extinction). The abundance of expanded (CTG)_n_ repeats in *DMPK* in the brain, which was reported to be one of the features of DMSXL strain, has been associated with short term memory impairments, anhedonic-like behaviour and neurochemical changes.^12^ The impact of these genetic changes on rule learning, however, has not yet been assessed. Reversal learning and extinction tasks performed in this study offer a way to analyze this in WT and DMSXL mice. Our study sought to evaluate the differences in reversal learning and extinction performances between DMSXL and WT mice and to interpret these findings in the light of the reported behavioral inflexibility in human DM1.

Our data suggest that both DMSXL and WT mice can switch and learn a new behavioral strategy, suggesting that cognitive flexibility is intact in both groups, as shown by similar correct response performances between DMSXL and WT mice in the early phase of reversal learning. It appears, however, that while both groups can successfully acquire the rule, DMSXL mice were able to automate it to a higher degree as shown by an increased percentage of correct touches in the late phase of reversal learning compared to WT mice. This has been suggested to indicate increased habit formation^21^ and may be interpreted as perseverative-like behaviour in DMSXL mice. If the mice make a mistake in their response, they have the opportunity to perform a correction session, *i.e.,* do it again. As such, the number of correction sessions is a measure of the number of mistakes made by an animal during a task. Neither correction sessions nor correction errors varied between the groups in the task acquisition and reversal phases. However, the total number of errors (*i.e.,* the number of mistakes made overall by the mice performing the task (and not just following correction) is lower in the DMSXL group compared to WT mice, suggesting that DMSXL mice make less mistakes when learning a new rule than WT mice.

The DMSXL mice post-reversal demonstrate a high level of behavioral perseveration compared to WT controls and suggest deficits in rule learning and switching. Perseverative behaviours are one of the prominent clinical features of OCD and autism which are also present in DM1 patients.^22^ As such they reflect cognitive inflexibility in affected individuals and validates these aspects of the clinical phenotype as also being present in DMSXL mice. To be clear our data suggest that DMSXL mice shown no difference in the early phase of reversal learning but perseverative behaviours in the late phase of reversal learning which may reflect reliance on habits formed.

Furthermore, the correct touch latencies after reversal differ between DMSXL and WT mice with DMSXL group taking significantly more time to respond overall and in the early phase of the reversal learning (sessions 1–4), whereas in the late phase (sessions 8–16) no such difference is observed. This may imply that DMSXL mice are more careful learning the task, therefore taking them increased amounts of time to make a choice. Once the task has been learned however, DMSXL mice are able to perform automated responses at similar latencies to WT, thereby indicating that the difference in the early phase is not related to motor impairment. (Moreover no gross motor deficits were seen in the DMSXL mice compared to WT controls.). There are no differences across sex in the correct touch latencies following reversal overall while in the early phase (sessions 1–4), females tend to be slower in making correct responses than their genotype matched male equivalent. Whether this represents more a greater degree of care in the females before making the response or a relatively higher degree of impulsivity in the males is not clear, but this is short-lived as no sex associated differences are seen when the reversal learning is sufficiently acquired. An alternative explanation for the data seen could be that the DMSXL mice are trading latency for accuracy on these tasks in the early phase; a strategy that may be an adaptation or even beneficial.

Interestingly, the latencies to collect the reward following reversal are higher in DMSXL mice compared to WT. This may imply a decrease in motivation to obtain the reward in DMSXL mice, as we have previously concluded this increase in latency is unlikely to be related to locomotion. Alternatively, DMSXL mice may be slower to turn: since the reward chamber is on the opposite side to the touchscreen of the chamber, mice must make an effort to turn 180 degrees away from touchscreen to collect the reward. It is possible that given the cerebellar synaptic plasticity deficits known to occur in DMSXL mice,^23^ that these mice have balance and motor coordination deficits which may impair their reward collection latency which was not observed on a gross motor coordination level. Clinically, it is known that impaired balance, stumbles and falls happen daily in DM1 patients, which may be associated with impaired cerebellum functioning.

In summary, DMSXL mice tend to perform just as well or better than WT controls in reversal learning (increased percentage of correct touches, reduced number of errors, similar correct touch latencies). The only differences in reversal learning were in slightly higher number of correct touches number in the late phase and a slower ability to collect rewards (which may reflect apathy-like behaviour) in DMSXL mice compared to their WT controls.

With regards to the appetitive extinction task, it is clear that the DMSXL mice show an increased number of blank touches and increased correct touch and rewards latencies. This may reflect that the DMSXL mice make more mistakes in the initial learning of this task and are also slower to make correct responses. Similar to the reversal learning task, they also demonstrate increased reward collection latency which may reflect a lack of motivation (apathy-like behaviour) to collect the reward. This lack of selectivity for the stimulus - reward relationship may also underlie the increased number of non-rewarded blank touches in DMSXL mice compared to controls.

The extinction task examines the act of decoupling a previously established stimulus - reward relationship, such that the stimulus is no longer rewarded. During extinction, DMSXL mice make fewer correct touches than WT mice but also make fewer correct touches very rapidly (already within the first few sessions) which means they rapidly stop a non-rewarded action. This suggests heightened sensitivity to the value of a reward.

The reduction in the number of correct touches made by the DMSXL mice compared to WT controls may also suggest that DMSXL mice are less motivated to make a correct response in the absence of a reward and can successfully inhibit a non-rewarded strategy. The increase in number of omissions in DMSXL mice also indicates that the DMSXL mice are unwilling to make any extra effort in the absence of reward, further supporting the idea of decreased motivation in these mice. A more thorough investigation of the motivation for reward in DMSXL mice could involve their testing on a progressive ratio schedule task in the future. This decrease in motivation, if confirmed, is similar to the apathy reported in DM1 patients.^24^ Moreover, no effect of sex was observed in any of the extinction learning paradigm metrics suggesting any differences are related to the genotype alone.

In our behavioral analysis, DMSXL mice are evidently slower in learning novel tasks, as observed by the latency increase to perform a correct action each time a new contingency is administered, compared to WT mice. The DMSXL mice show an increased latency to perform an action following a change in contingency. This has also been reported by others in the context of novelty-induced inhibition in the open field in DMSXL mice, suggestive of increased anxiety.^12^ This freezing / slower response behaviour when challenged with an unfamiliar environment / stimulus is also seen in the visual discrimination reversal and extinction paradigms as well as in the first few sessions of all new contingencies.

In both reversal learning and extinction tasks, it is clear that DMSXL mice show increased reward collection latencies across both tasks suggestive of decreased motivation compared to their WT controls. This is important as it shows a decreased incentive to perform an action for a sweetened reward. This is in concurrence with earlier data reporting differences in the saccharine preference test between DMSXL and WT mice in which DMSXL mice consumed less saccharine compared to WT mice which is a proxy of anhedonia like behaviour. The sensitivity for saccharine sweetened reward was less in the DMSXL mice compared to WT controls.^12^ With regard to rule learning and rule switching, DMSXL mice are slower to learn but perform better in reversal learning and appetitive extinction compared to WT controls. Only an increased number of correct touches in the late phase of reversal learning may reflect perseverative behaviour. Taken together, this suggests that the DMSXL mouse does not necessarily reflect the reported OCD and autistic traits seen in clinical DM1 groups but may reflect the apathy seen in these patients. Initial learning deficits observed in the DMSXL mouse model may reflect similar behavioral phenotypes seen in DM1 such as cognition and motor learning impairments. Homozygous DMSXL mice reflect more the congenital form of DM1 than the adult form.^25^ The further validation of the DMSXL model is warranted in particular to evaluate the motivational aspects of the DM1 phenotype Additional behavioural testing with confirmatory experiments to extend the findings of this pilot study would be prudent. In particular, expanding the studies with an increased number of animals (to fully assess sex differences), and explore the motivation and other cognitive findings in these studies with confirmatory tests such as the progressive ratio schedule task. Ideally an interventional test using an anti-sense oligonucleotide against DMPK would also be included in order to evaluate whether these cognition and motivational aspects change with decreased DPMK expression. The inclusion of additional controls including the parental lines with smaller CTG repeat numbers in mutant DMPK would be helpful as well to help build confidence that the findings are attributable to the CTG repeat expansions and not the insertion site of the transgene *per se*. Both depression (which includes apathy as one of its symptoms) and obsessive-compulsive traits have been investigated in a number of genome-wide association studies (GWAS) focusing on single nucleotide polymorphisms (SNPs).^26,27^ In two studies, both depression and obsessive-compulsive phenotype are strongly associated with SNPs within the sequences encoding elements of insulin-resistance which may reflect pro-inflammatory states.^28,29^ A recent review has highlighted a potential role for insulin signaling in DM1.^30^ Whether any of the behavioral changes reported here, relate to altered insulin resistance or downstream pro-inflammatory status merits further investigation.

Furthermore, it would be interesting to characterize the molecular mechanisms underlying the motivational changes in the phenotype of the DMSXL mice in order to evaluate whether strategies targeting them could rescue the ‘apathy-like’ behavioral phenotype documented here. In this regard, the study to identify transcriptomic and protein markers in brain regions involved in motivation and reward valuation, *e.g.,* cingulate and insular cortices, nucleus accumbens and cerebellum, would be a possible next step. In addition, while insulin resistance modifying drugs such as metformin are reported to positively impact on DM1 motor symptoms in patients,^31^ the study of metformin on motivation in DMSXL mice is missing. This would be a useful next step in addition to considering more novel drugs affecting motivation such as GLP-1 agonists. Care would need to be exerted here as these agents are typically deployed for weight loss and DMSXL mice have a lower body weight than their age matched WT controls.

**Abbreviations**
DM1: Myotonic dystrophy type I
*DMPK*
: dystrophia myotonica-protein kinase
OCD: obsessive compulsive disorder;
VDR: visual discrimination and reversal;
DMSXL: human *DMPK* CTG repeat expansion (>1500 CGT) transgenic mouse model
WT: wild type
AUC: area under the curve
CNS: central nerve system
ns: not significant
s: seconds

## Supplemental Material

sj-docx-1-jnd-10.1177_22143602251339350 - Supplemental material for Altered reversal and extinction learning in the DMSXL mouse model of type I myotonic dystrophy (DM1): An exploratory study Supplemental material, sj-docx-1-jnd-10.1177_22143602251339350 for Altered reversal and extinction learning in the DMSXL mouse model of type I myotonic dystrophy (DM1): An exploratory study by Sylvia Nieuwenhuis, Denys Kozakov, Kasia Kapusta, Geneviève Gourdon and Jeffrey C Glennon in Journal of Neuromuscular Diseases
