# Supplementary material for: Altered reversal and extinction learning in the DMSXL mouse model of type I myotonic dystrophy (DM1): An exploratory study
Source: J Neuromuscul Dis. 2025 May 22;12(4):535–47. doi: 10.1177/22143602251339350 (PMC13142853; doi:10.1177/22143602251339350)
Supplement: sj-docx-1-jnd-10.1177_22143602251339350 - Supplemental material for Altered reversal and extinction learning in the DMSXL mouse model of type I myotonic dystrophy (DM1): An exploratory study [file sj-docx-1-jnd-10.1177_22143602251339350.docx]

Table 1: Bonferroni-corrected independent t-test results from WT versus DMSXL mice for correct touches in reversal learning test (late phase, session 1-16). Data is presented both for nominal significance (non-adjusted two-tailed p-values) and after Bonferroni-correction.


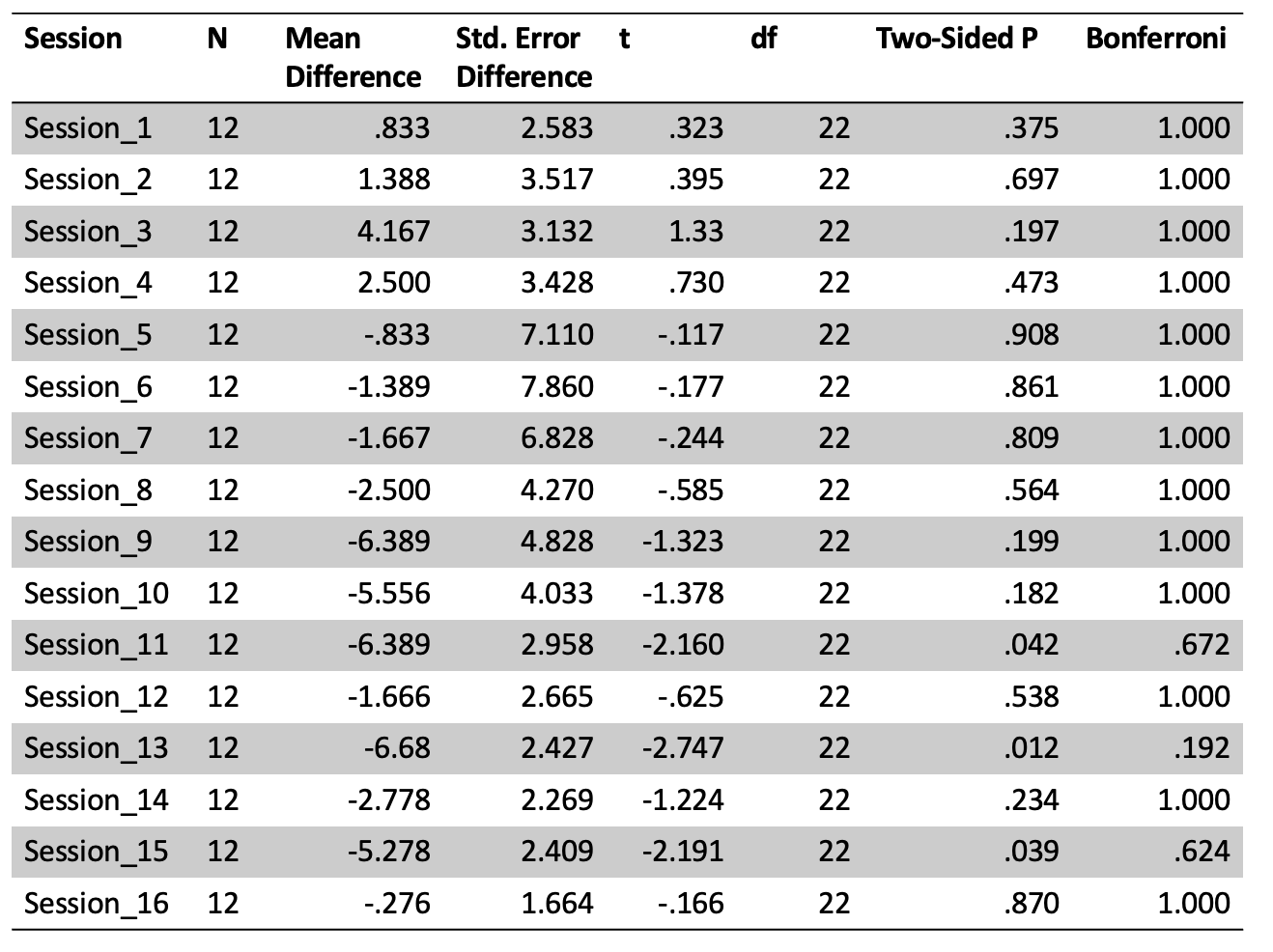


**Table 2:** Breakdown of the different types of responses and percentages, including the total number of sessions.
